# Virus-like particles displaying the mature C-terminal domain of filamentous hemagglutinin are immunogenic and protective against *Bordetella pertussis* respiratory infection in mice

**DOI:** 10.1128/iai.00270-24

**Published:** 2024-07-18

**Authors:** Gage M. Pyles, Annalisa B. Huckaby, Maria de la Paz Gutierrez, William T. Witt, Margalida Mateu-Borrás, Spencer R. Dublin, Carleena Rocuskie-Marker, Bethany N. Sesti, Kerrington Peasak, Graham J. Bitzer, Nathaniel Rader, Kelly L. Weaver, Dylan T. Boehm, Nicholas Fitzgerald, Joshua Chapman, Samuel Ulicny, F. Heath Damron, Mariette Barbier

**Affiliations:** 1 Department of Microbiology, Immunology and Cell Biology, West Virginia University, Morgantown, West Virginia, USA; 2 Vaccine Development Center, West Virginia University Health Sciences Center, Morgantown, West Virginia, USA; University of California, Davis, Davis, California, USA

**Keywords:** *Bordetella pertussis*, whooping cough, pertussis, DTaP, pertussis toxin, VLP, FHA, vaccines, bacterial challenge, pertussis mouse model, vaccine evaluation

## Abstract

*Bordetella pertussis,* the bacterium responsible for whooping cough, remains a significant public health challenge despite the existing licensed pertussis vaccines. Current acellular pertussis vaccines, though having favorable reactogenicity and efficacy profiles, involve complex and costly production processes. In addition, acellular vaccines have functional challenges such as short-lasting duration of immunity and limited antigen coverage. Filamentous hemagglutinin (FHA) is an adhesin of *B. pertussis* that is included in all multivalent pertussis vaccine formulations. Antibodies to FHA have been shown to prevent bacterial attachment to respiratory epithelial cells, and T cell responses to FHA facilitate cell-mediated immunity. In this study, FHA’s mature C-terminal domain (MCD) was evaluated as a novel vaccine antigen. MCD was conjugated to virus-like particles via SpyTag-SpyCatcher technology. Prime-boost vaccine studies were performed in mice to characterize immunogenicity and protection against the intranasal *B. pertussis* challenge. MCD-SpyVLP was more immunogenic than SpyTag-MCD antigen alone, and in Tohama I strain challenge studies, improved protection against challenge was observed in the lungs at day 3 and in the trachea and nasal wash at day 7 post-challenge. Furthermore, a *B. pertussis* strain encoding genetically inactivated pertussis toxin was used to evaluate MCD-SpyVLP vaccine immunity. Mice vaccinated with MCD-SpyVLP had significantly lower respiratory bacterial burden at both days 3 and 7 post-challenge compared to mock-vaccinated animals. Overall, these data support the use of SpyTag-SpyCatcher VLPs as a platform for use in vaccine development against *B. pertussis* and other pathogens.

## INTRODUCTION

*Bordetella pertussis* is the Gram-negative bacterium responsible for the respiratory disease whooping cough, or pertussis (1). Although pertussis affects all age groups, it is most severe in infants too young to be vaccinated and remains a global health concern with more than 60,000 reported cases worldwide in 2022 (2). Current vaccines for pertussis consist of whole-cell or acellular subunit vaccines (3). Whole-cell pertussis (wP) vaccines are cost-effective and easily producible but are associated with adverse side effects, including prolonged and inconsolable crying, fever, and febrile seizures (4). Acellular pertussis (aP) vaccines are also widely used and contain antigens produced and purified from *B. pertussis* including pertussis toxin (PT), pertactin, fimbriae 2/3, and filamentous hemagglutinin (FHA).

In the US, wP vaccines were phased out and replaced with aP vaccines in 1996, whereas countries such as Japan replaced wP vaccines as far back as the 1980s (3, 5, 6). One of the barriers associated with the broader use of aP vaccines in developing countries is the relatively high cost of aP vaccine production (7). The production of aPs is a highly complex and resource-intensive process that begins with the culture of *B. pertussis*. Specialized media are used to promote the expression of vaccine antigens during culture, and downstream, these antigens are subjected to a meticulous purification process involving precipitation, filtration, and chromatographic techniques (8 – 12). Furthermore, antigens also undergo chemical detoxification to eliminate pertussis toxin activity and improve stability, resulting in diminished epitope recognition (13 – 15). Each stage of this process undergoes extensive quality control measures to ensure standardization and purity of antigens before vaccine formulation and lot validation (16).

Of the antigens included in aPs, FHA plays a crucial role in *B. pertussis* pathogenesis as one of the primary adhesins that enables binding to host cells, facilitating infection (17 – 20). FHA is one of the first virulence factors produced upon infection and is initially translated as an ~370 kDa polypeptide (19, 21). FHA is then cleaved at its C-terminus during export to become the mature ~220 kDa protein that is transiently bound to the surface of *B. pertussis* and later released into the extracellular milieu (19, 22). The 220 kDa secreted form of FHA is the antigen currently included in aP vaccines (23). Interestingly, the mature C-terminal domain (MCD) of FHA, which spans the latter 500 amino acids of this protein, is one of the most immunodominant regions of FHA, as evidenced by robust antibody responses in vaccinated and/or infected individuals (24 – 26). Additionally, antibodies that bind to MCD rather than the β-helix of FHA inhibit the adherence of *B. pertussis* and *Bordetella bronchiseptica* to epithelial and macrophage-like cells *in vitro*, suggesting a potential role of anti-MCD antibodies in eliciting protective immunity against pertussis (27). This is supported by studies demonstrating that truncated forms of FHA, which include portions of the MCD-coding sequence, can be protective antigens against *B. pertussis* challenge in mice (27 – 29). Additionally, MCD antigens can be produced recombinantly, which would reduce the cost of large-scale antigen purification for aP vaccines.

We hypothesize that it is possible to use a truncated FHA antigen comprising the MCD as a replacement for the full-length FHA antigen currently used in pertussis acellular vaccines. To test our hypothesis, a truncated FHA molecule conjugated to virus-like particles (VLPs) using SpyTag-SpyCatcher technology was evaluated as a vaccine antigen (30). VLPs offer an alternative method for generating effective vaccines and have expanded in popularity alongside recent mRNA-based and viral-vector-based vaccines. Compared to subunit vaccines, VLPs have proven more immunogenic due to their presentation of dense, repetitive epitopes, which elicit strong T and B cell responses (31). This platform has previously shown high thermostability and has been effective in displaying antigens from various pathogens as well as cancers (32 – 35).

In this study, MCD-SpyVLP was shown to be highly immunogenic and led to the production of antibodies that bind to both FHA and *B. pertussis in vitro*. As a non-conjugated single-antigen vaccine, SpyTag-MCD confers significant but incomplete protection against *B. pertussis* in mice. Furthermore, vaccine efficacy was enhanced against a strain of *B. pertussis* lacking PT activity (due to genetic inactivation). When conjugated to SpyVLPs, both immunogenicity and protection were significantly enhanced against either strain of *B. pertussis*. Overall, these data support the potential use of MCD as a vaccine antigen, and SpyVLPs as a vaccine delivery platform for next generation pertussis vaccines.

## MATERIALS AND METHODS

### *B. pertussis* strains and growth

Wild-type *B. pertussis* strain Tohama I and Tohama I PT^mut^ were generously provided by Dr. Peter Sebo at The Czech Academy of Sciences. Tohama I PT^mut^ was constructed using allelic exchange as described previously (36). *B. pertussis* strain UT25Sm1 was kindly provided by Dr. Sandra Armstrong (University of Minnesota). *B. pertussis* strains were grown as described previously (37 – 39). Briefly, strains were cultured on Bordet-Gengou agar (VWR, Cat. #90003-41) supplemented with 15% defibrinated sheep’s blood (Hemostat Laboratories, Cat. #DSB500) and 40 µg/mL cephalexin (Sigma-Aldrich, Cat. #C4895) for 48 h at 36°C. *B. pertussis* was then collected using sterile polyester swabs (Puritan, Cat. #22-029-574) and transferred into 20 mL of sterile Stainer-Scholte liquid media in 125 mL flasks at 36°C with constant agitation at 180 rpm until reaching an OD_600nm_ with 1 cm path width of 0.4–0.5 before subsequent use (10). For use in enzyme-linked immunosorbent assay (ELISA) or intranasal challenge, liquid cultures were diluted to an OD_600nm_ of 0.245 corresponding to 10^9^ CFU/mL, before further dilution and use.

### Recombinant antigen and MCD-SpyVLP production

SpyCatcher003-mi3 particles were kindly provided by the Howarth lab at the University of Cambridge. SpyTag-MCD was expressed using a pET30a vector in *Escherichia coli* by GenScript and purified using sequential nickel affinity chromatography and Q-column cation exchange chromatography. The SpyTag-MCD construct used in this study encompasses amino acids 1,871–2,362 of full-length FhaB with the addition of an N-terminal hexahistidine tag and SpyTag. Samples were treated to remove excess endotoxin, yielding a final endotoxin concentration of ≤1.4 EU/mg SpyTag-MCD. SpyTag-SpyCatcher VLP conjugations were performed as described in reference (40). Briefly, MCD-SpyVLP was conjugated at a ratio of 5.1 µg SpyTag-MCD to 2 µg SpyVLP overnight at 4°C with end-over-end rotation in tris-buffered-saline, pH 8.0. Samples from each conjugation were mixed with 4× Laemmli buffer (Bio-Rad, Cat. #1610747), heated to 95°C for 10 min, and resolved in Any kD Mini-PROTEAN TGX stain-free precast gels (Bio-Rad, Cat. #4569033) at 200 V for 30 min. Gels were then stained with Blazin’ Blue protein gel stain (Gold Biotechnology, Cat. #P-810-1) for 1 h and destained overnight at room temperature prior to imaging using a Bio-Rad Chemidoc Touch imaging system on auto-optimal settings.

### Electron microscopy

MCD-SpyVLP samples were prepared as previously described. After conjugation, VLPs were applied to UV-treated, carbon-coated EM grids (Ted Pella 01843-F) and stained with uranyl acetate (1% aqueous). Micrographs were recorded at 100,000× magnification on a JEOL 1010 microscope equipped with an ATM Hamamatsu Orca-HR digital camera.

### Vaccine formulation and immunization

Non-conjugated SpyTag-MCD or conjugated MCD-SpyVLP at 1.25 µg/50 µL doses were adsorbed to 3.125 µg of Aluminum hydroxide (Alhydrogel) (Invivogen, Cat. #Vac-Alu-250) with end-over-end rotation 1 h prior to immunization. Separate sets of 5-week-old outbred female CD-1 mice (Charles River) were then vaccinated with SpyVLP-only (vehicle), or either MCD formulation twice, with a 3-week interval between prime and boost.

### Serological analysis of antibodies

Serum antibody responses to FHA and *B. pertussis* were quantified using enzyme-linked immunosorbent assay. High-binding, 96-well microtiter plates (ThermoFisher, Cat. #15041) were coated with 50 µL/well of 1 µg/mL FHA (The Native Antigen Company, BP-FHA-100) in phosphate-buffered saline (PBS) or *B. pertussis* strain UT25Sm1 to an OD_600nm_ of 0.245 (10^9^ CFU/mL) overnight at 4°C. After coating, the plates were blocked overnight using 200 µL/well of 5% nonfat dry milk (Nestle Carnation, Cat. #00500002292840) in PBS-tween 20 (PBS-T) (Sigma Aldrich, Cat. #P1379-1L). The plates were then washed with PBS-T, and sera were prepared at an initial dilution of 1:1,000 in 5% nonfat dry milk in PBS-T. All serum samples were then diluted 1:2 across the microtiter plates to a final concentration of 1:16,384,000 and incubated for 2 h at 37°C. Plates were then washed and incubated with 100 µL/well goat anti-mouse IgG horse-radish peroxidase-conjugated (HRP) antibodies (Novus Biologicals, Cat. #NBP1-75130) and diluted 1:2,000 for 1 h at 37°C. Plates were then washed and incubated with 100 µL of tetramethylbenzidine (TMB) substrate (BioLegend, Cat. #421101) for 30 min at room temperature covered from light. After 30 min, 50 µL of 2 M sulfuric acid (Fisher Scientific, Cat. #SA818500) was added to stop the reaction. Absorbance at OD_450 nm_ was then read using a SpectraMax i3 plate reader (Molecular Devices). The lower limit of detection for all ELISAs was set at the starting serum dilution of 1:1,000, and serum titers were determined as the final dilution, which had an absorbance greater than or equal to twice that of negative controls.

### Peptide epitope mapping

BepiPred 3.0 was utilized leading to the identification of 10 peptides within MCD as potential linear B cell epitopes. These peptides were synthesized with an N-terminal biotin SGSG linker (ThermoFisher) to allow for compatibility in ELISA. Each peptide was received as a lyophilized powder and reconstituted in 50% DMSO to a final concentration of 1 mg/mL. Pre-blocked streptavidin-coated plates (Pierce, Cat. #15125) were coated with 50 µL of each biotinylated peptide diluted 1:1,000 in PBS overnight. Plates were washed with PBS-T, and 50 µL of pooled sera diluted 1:100 was added overnight. Plates were then washed with PBS-T, and 100 µL of goat anti-mouse IgG-HRP antibodies diluted 1:2,000 was added to each plate for 1 h at 37°C. Plates were washed, and 100 µL of TMB substrate was added to each well. Plates were incubated for 30 min at room temperature before the addition of 50 µL of 2 N sulfuric acid to each well to stop the reaction. The absorbance at OD_450 nm_ of each well was read using a SpectraMax i3 plate reader, and the change in absorbance in comparison to wells with sera from vehicle-immunized mice was calculated and used to quantify antibody binding to each peptide.

### *B. pertussis* challenge, euthanasia, and bacterial burden quantification

Murine challenge with *B. pertussis* was performed as previously described in references (37 – 39). Briefly, 2 weeks post-boost, mice were anesthetized via intraperitoneal injection of 7.7 mg ketamine/kg of body weight (Patterson Veterinary, Cat. #07-803-6637) with 0.77 mg xylazine/kg of body weight (Patterson Veterinary, Cat. #07-808-1939) in sterile 0.9%, wt/vol NaCl (Baxter, Cat. #2F7124). Mice were then challenged intranasally (10 µL/nostril) with 2 × 10^6^ CFU of *B. pertussis* Tohama I or Tohama I PT^mut^. Separate sets of mice were then euthanized via intraperitoneal injection of Euthasol (390 mg pentobarbital sodium/kg of body weight) (Patterson Veterinary, Cat. #07-805-9296) in sterile 0.9%, wt/vol NaCl at days 3 or 7 post-challenge. Following euthanasia, lungs and trachea were collected and homogenized in 1 mL of sterile PBS using a Polytron PT 2500 E homogenizer (Kinematica). Additionally, nasal wash samples were collected post-mortem by flushing 1 mL of sterile PBS through the nares at the base of the neck into sterile 1.5 mL Eppendorf tubes. All samples were then serially diluted 10-fold in PBS and plated on BG agar containing 15% defibrinated sheep’s blood and 40 µg/mL cephalexin. Plates were then incubated at 36°C for 72 h, and CFUs were enumerated and normalized to CFU/mL.

### Neutrophil enumeration

Blood was collected post-euthanasia via cardiac puncture into BD Microtainer blood collection tubes containing K_2_EDTA (BD, Cat#365974). The number of neutrophils present in whole blood samples was measured using a ProCyte Dx Hematology Analyzer (IDEXX).

### ELISpot sample preparation and analysis

Mouse IgG Single-Color ELISpot Kits (Immunospot) were used to quantify anti-FHA antibody-secreting cells (ASCs) in the bone marrow of mice 2 weeks after their second immunization. Briefly, PVDF membrane 96-well plates were coated with 80 µL of 1 µg/mL FHA overnight per the manufacturer’s instructions. Bone marrow was collected 2 weeks post-boost following euthanasia by flushing each side of the left hind femur with 0.5 mL of CTL test medium (Immunospot, Cat. #CTLT-005) containing L-glutamine (Sigma-Aldrich, Cat. #G8540). After flushing, cells were run through 70 µm filters (BioDesign, Cat. #N70R), washed once with 0.3 mL of the same media, and centrifuged at 400 × *g* for 4 min at 4°C. Cells were then resuspended in 1 mL of fetal bovine serum (Gibco, Cat. #10437028) containing 10% dimethyl sulfoxide (ThermoFisher, Cat. #BP231-100) and frozen at −80°C until use. Upon analysis, bone marrow cells were thawed at 37°C in a water bath, washed once with 1 mL of sterile, endotoxin-free PBS (MilliporeSigma, Cat. #TMS012A), and resuspended in 1 mL CTL culture medium. Cells were enumerated by mixing 1:1 with trypan blue stain and counted on a Countess III Automated Cell Counter (ThermoFisher). Plates were then washed with PBS, and cells were added and serially diluted twofold for three dilutions. Cells were then incubated overnight at 36°C, and plates were developed according to the manufacturer’s protocol. Dilutions with spots ranging from 10 to 100 were used to quantify anti-FHA antibody-secreting cells and were normalized to spots per 10^6^ cells.

### Passive immunization with immune sera

Passive immunization was performed as previously described with slight modifications (41). Briefly, sera were collected for passive immunization experiments from separate groups of mice vaccinated with SpyVLP-only (vehicle), 1/20th human doses of DTaP Infanrix, SpyTag-MCD, or MCD-SpyVLP as previously described. Two weeks post-boost, serum was collected via cardiac puncture following euthanasia. Sera were pooled before administering 250 µL per mouse intraperitoneally 1 h before the challenge with *B. pertussis* as previously described.

### Endotoxin quantification

Endotoxin present in samples of vaccine antigen was quantified using a Pierce Chromogenic Endotoxin Quantification Kit (ThermoFisher, Cat. #A39552S) following the manufacturer’s protocols. Antigen samples were serially diluted 10-fold to ensure reaching endotoxin standard ranges of 1–0.1 EU/mL and were analyzed in triplicate.

### Statistics

Statistical analyses were performed using Prism version 10 software (GraphPad). For comparisons between two groups, unpaired *t*-tests were performed. Data sets comprising three or more groups were analyzed using one-way analysis of variance (ANOVA) followed by Tukey’s multiple comparison test for normally distributed data. All data sets containing log-normally distributed data were log-transformed prior to statistical analysis. Non-parametric data were analyzed using the Kruskal-Wallis test with Dunnett’s *post hoc* test.

## RESULTS

### SpyCatcher003-mi3 virus-like particles can be used to display SpyTag-MCD

**Fig 1:**
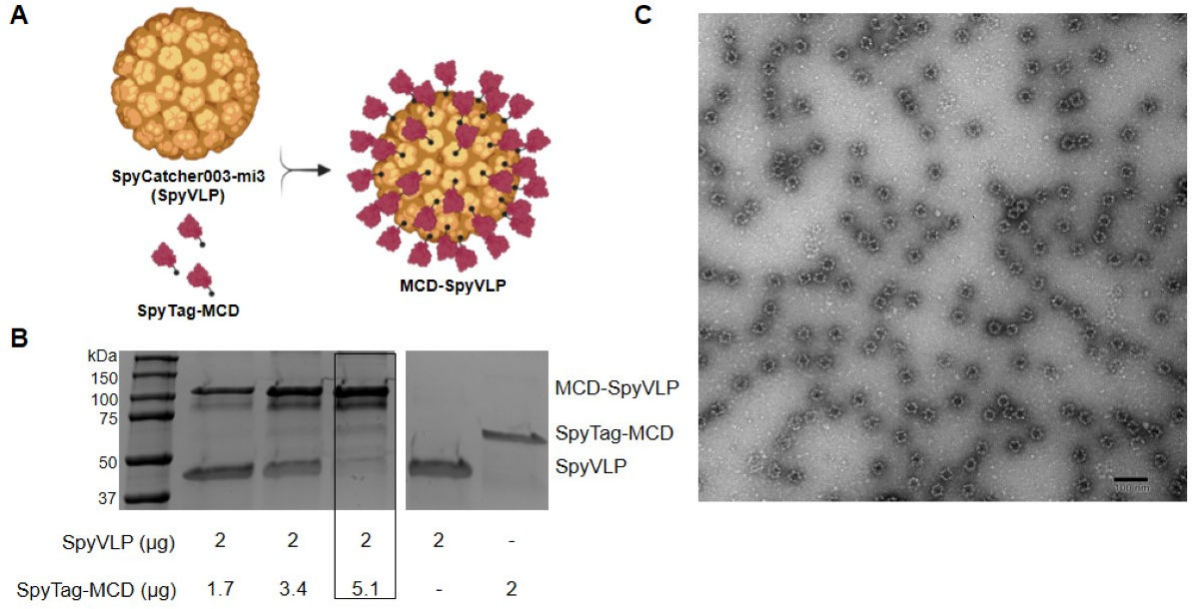
SpyCatcher003-mi3 virus-like particles can be used to display SpyTag-MCD. (**A**) Schematic representation of MCD-SpyVLP assembly. The VLP is formed by the self-assembly of SpyCatcher003-mi3 monomers into a 60-mer capsid-like structure. SpyTag-MCD molecules are covalently bound to each SpyVLP subunit via SpyTag-SpyCatcher interactions to form MCD-SpyVLP. (**B**) SDS-PAGE analysis of MCD-SpyVLP conjugation efficiency. SpyTag-MCD and SpyVLP were mixed at increasing ratios of SpyTag-MCD:SpyVLP to determine the optimal ratio at which low levels of free SpyTag-MCD and SpyVLP remain. For vaccinations, the outlined reaction ratio was used. (**C**) Representative electron micrograph of MCD-SpyVLPs taken at 100,000× magnification (scale bar: 100 nm).

The first objective of this study was to design a soluble and immunogenic polypeptide antigen that retains the protective characteristics of FHA. To do this, the design focused on a 56-kDa region encompassing amino acids 1,871–2,362 of FhaB corresponding to the MCD region. This region was selected based on high immunogenicity and solubility (25, 28). To facilitate conjugation to virus-like particles using SpyTag-SpyCatcher technology, an additional 13 amino acid (AHIVMVDAYKPTK) SpyTag was added onto the N-terminus of MCD. The SpyCatcher003-mi3 VLPs used for conjugation comprised an engineered aldolase protein (~45 kDa/subunit), which spontaneously self-assembles into a dodecahedral 60 subunit protein cage (42). Each subunit within the cage is translationally fused to the SpyCatcher003-mi3 protein, which can be subsequently conjugated to antigens labeled with a SpyTag via irreversible, covalent iso-peptide bonds, allowing for the presentation of approximately 60 antigens per particle. (Fig. 1A) (32). The antigen fusion containing the SpyTag and amino acids 1,871–2,362 of FhaB is herein referred to as SpyTag-MCD. To produce this vaccine antigen, the ideal conjugation ratio of SpyTag-MCD to SpyVLP was determined by mixing increasing amounts of SpyTag-MCD with constant amounts of SpyCatcher003, followed by SDS-PAGE analysis (Fig. 1B). A 2.55:1 mass-to-mass SpyTag-MCD to SpyCatcher003-mi3 conjugation ratio was selected for the conjugation of 5.1 µg of SpyTag-MCD per 2 µg of SpyVLP, resulting in effective conjugation with little to no free SpyTag-MCD or SpyCatcher003-mi3 remaining (Fig. 1B). Successful conjugation with the formation of visible VLPs was confirmed via electron microscopy (Fig. 1C). The SpyCatcher003-mi3 particles labeled with SpyTag-MCD are herein referred to as MCD-SpyVLP. Collectively, these data demonstrate the successful assembly of the VLP-based MCD antigen, and the immunogenicity of MCD-SpyVLP was next evaluated.

### Conjugation to SpyCatcher003-mi3 VLPs increases SpyTag-MCD immunogenicity

**Fig 2:**
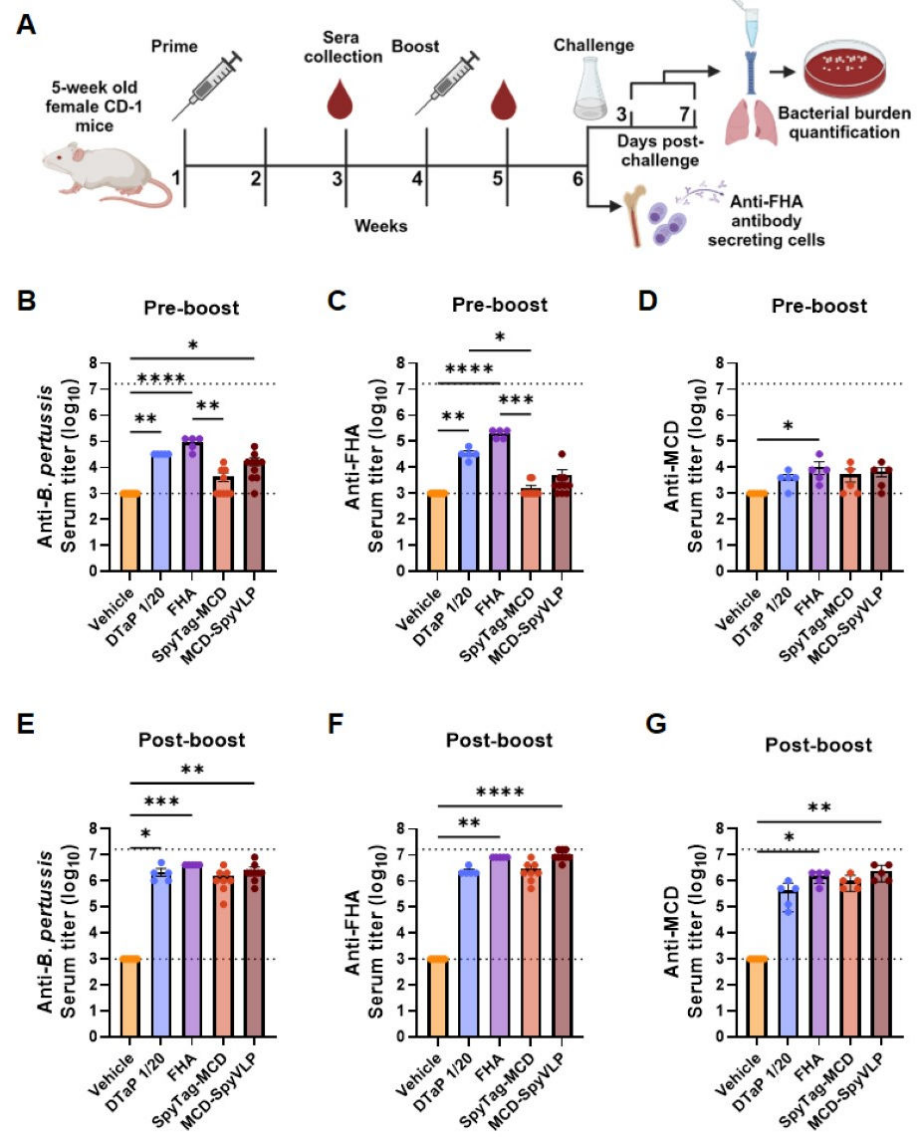
Conjugation to SpyCatcher003-mi3 VLPs increases SpyTag-MCD immunogenicity. (**A**) Five-week-old CD-1 female mice were primed and boosted with the vehicle, SpyTag-MCD, or MCD-SpyVLP 3 weeks apart at weeks 1 and 4. Sera were collected via submandibular bleeding 1 week before and after boost. Two weeks post-boost, separate sets of mice were euthanized for bone marrow collection and quantification of anti-FHA antibody-secreting cells or challenged intranasally with 2 × 10^6^ CFU of *B. pertussis* Tohama I. After the challenge, separate sets of mice were euthanized at day 3 or 7 for bacterial burden quantification in the lungs, trachea, or nasal lavage. Serum IgG titers against *B. pertussis* (**B and E**)*,* FHA (**C and F**), and MCD (**D and G**) 1 week before (**B, C, and D**) or after (**E, F, and and G**) boost. *P*-values were calculated using Kruskal-Wallis test with Dunnett’s *post hoc* test due to these data being non-parametric, *P < 0.05 and **P < 0.01 (*n* = 5–10 mice per group, bars represent mean ± SEM).

To determine the immunogenicity of SpyTag-MCD and MCD-SpyVLP, female CD-1 outbred mice were immunized to perform serological studies as depicted in Fig. 2A. Based on previous studies in our lab, 1/20th of the Infanrix aP human dose (containing 1.25 µg FHA) was selected as a positive control for protection against *B. pertussis* in murine studies (37). To allow for comparison between FHA, SpyTag-MCD, and MCD-SpyVLP, both antigens were formulated to contain 1.25 µg of SpyTag-MCD antigen per dose. In addition, all vaccines were formulated with aluminum hydroxide (alum) as an adjuvant at an equivalent mass to that contained in 1/20th of the Infanrix aP human dose (31.25 µg). The negative control group was immunized with SpyVLP (vehicle) with alum. Mice were first immunized at 5 weeks of age and boosted 3 weeks later with the same formulations. Serum was collected 1 week before and after booster to quantify anti*-B*. *pertussis*, anti-FHA, and anti-MCD serum IgG levels. After prime, only DTaP- and FHA-immunized mice had significantly elevated serum IgG levels against both *B. pertussis* and FHA compared to mock-immunized animals, while FHA-immunized mice also had greater antibody levels against MCD (Fig. 2B through D). Mice immunized with MCD-SpyVLP but not SpyTag-MCD had increased anti-*B*. *pertussis* serum IgG levels compared to mock-immunized mice after prime. After the boost, mice immunized with DTaP, FHA, and MCD-SpyVLP had increased anti-*B*. *pertussis* IgG levels compared to mock-immunized mice (Fig. 2E). Only FHA and MCD-SpyVLP vaccination led to a significant increase in anti-FHA and MCD antibody titer after a second dose (Fig. 2F and G). Altogether, these data demonstrate that MCD immunogenicity can be improved by conjugation to SpyCatcher003-mi3. In addition, the magnitude of the antibody response to MCD-SpyVLP resembles that of the antibody response following FHA vaccination.

### Peptide epitopes within MCD are differentially recognized in response to vaccination with DTaP, FHA, SpyTag-MCD or MCD-SpyVLP

**Fig 3:**
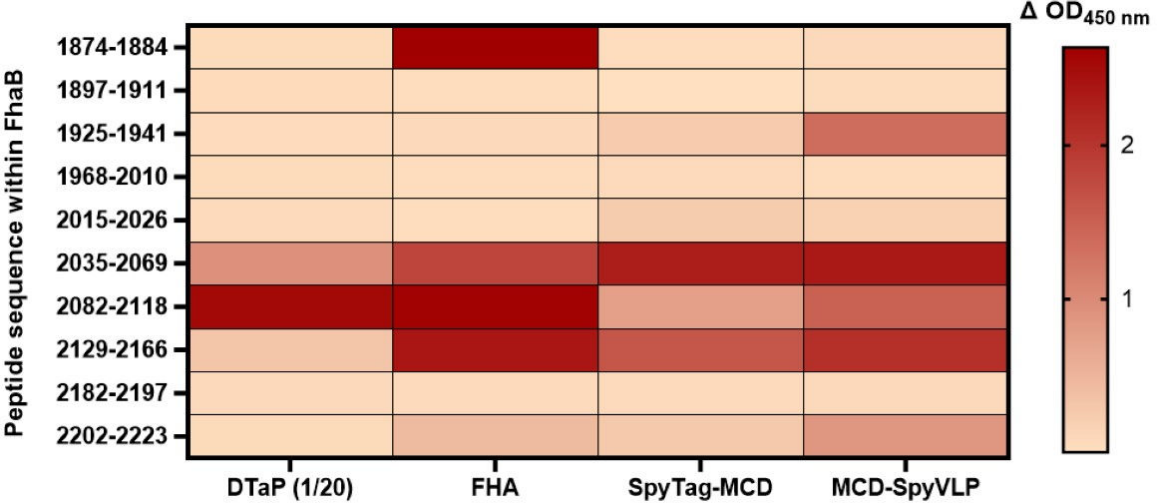
Peptide epitopes within MCD are differentially recognized in response to vaccination with DTaP, FHA, SpyTag-MCD, or MCD-SpyVLP. ELISA was used to quantify the binding of sera pooled from mice (*n* = 5) immunized with 1/20th human dose of DTaP, FHA, SpyTag-MCD, or MCD-SpyVLP to 10 linear peptides within the MCD region of FhaB. Results are represented as a heatmap of changes in absorbance values (ΔOD_450 nm_) from each group compared to vehicle-immunized mice.

To determine immunodominant regions within MCD that are recognized in response to vaccination with DTaP, native FHA, SpyTag-MCD, or MCD-SpyVLP, 10 linear peptides were synthesized which span amino acids 1,874–2,223 of FhaB. These peptides were selected due to their potential as linear B-cell epitopes based on BepiPred 3.0 predictions. Each peptide was modified with the addition of an N-terminal biotin linker and were immobilized on streptavidin-coated plates to enable their use in ELISA. Pooled sera from mice immunized with SpyVLP, DTaP, FHA, SpyTag-MCD, or MCD-SpyVLP were then added to the peptides to quantify antibody binding. Increases in antibody binding, depicted as ΔOD_450 nm_ over vehicle-immunized mice sera are shown in Fig. 3. Peptides spanning residues 2,035–2,069 and 2,082–2,118 are immunodominant among each vaccine. Interestingly, vaccination with SpyTag-MCD or MCD-SpyVLP led to a greater antibody response to peptide 2,129–2,166, which is recognized in response to native FHA but not DTaP, suggesting this epitope may be lost due to the DTaP detoxification process. Of note, the antibody response to peptides 1,925–1,941 and 2,202–2,223 was also greater after vaccination with FHA and MCD-SpyVLP compared to both DTaP and SpyTag-MCD. Altogether, these results show that each vaccine elicits distinct antibody-binding patterns toward MCD, while MCD-SpyVLP elicits the most similar binding pattern to native FHA. Overall, the humoral response to MCD-SpyVLP had the greatest breadth of binding to these peptides compared to DTaP and SpyTag-MCD.

### Vaccination with MCD-SpyVLP antigen leads to increased antigen-specific antibody-secreting cells in the bone marrow

**Fig 4:**
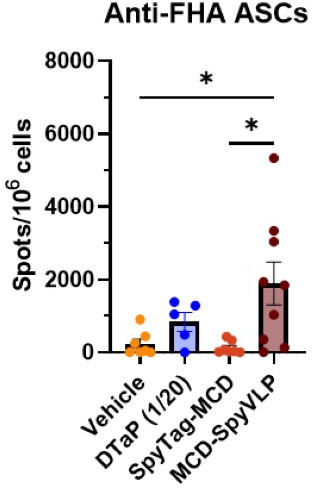
Vaccination with MCD-SpyVLPs leads to the early presence of antigen-specific antibody-secreting cells in the bone marrow. ELISpot quantification of anti-FHA ASCs in the bone marrow 2-weeks post-boost with SpyVLPs (vehicle), 1/20th DTaP, SpyTag-MCD, or MCD-SpyVLP. *P*-values were calculated using ordinary one-way ANOVA with Tukey’s multiple comparison test. *P < 0.05 (*n* = 5–9 mice per group, bars represent mean ± SD).

Understanding the persistence and efficacy of vaccine-induced immunity against *B. pertussis* is of paramount importance in the development of next-generation pertussis vaccines. Although there are currently no established parameters for measuring vaccine longevity, antibody-secreting cells in the bone marrow play a direct role in sustained antibody secretion over time (43). Additionally, in previous studies, ASCs were shown to be an immunological correlate of pertussis vaccine longevity and are increased in response to whole-cell pertussis vaccines associated with longer-lived immunity compared to DTaP (38). Thus, detecting the presence of these cells may better gauge the duration of the immune response and complement traditional methods such as measuring serum antibody levels. For this reason, a set of non-challenged mice was euthanized 2 weeks post-boost and anti-FHA ASCs in the bone marrow were quantified via ELISpot. In this experiment, mice vaccinated with MCD-SpyVLP had significantly higher numbers of anti-FHA ASCs 2 weeks post-boost than mice vaccinated with the vehicle control or SpyTag-MCD (Fig. 4). This suggests that while SpyTag-MCD is sufficient to generate antibody responses against *B. pertussis*, it is insufficient to generate the production of ASCs, which is enhanced by SpyVLP conjugation.

### Vaccination with MCD-SpyVLP facilitates control of bacterial burden in the airways of mice challenged with *B. pertussis* strain Tohama I

**Fig 5:**
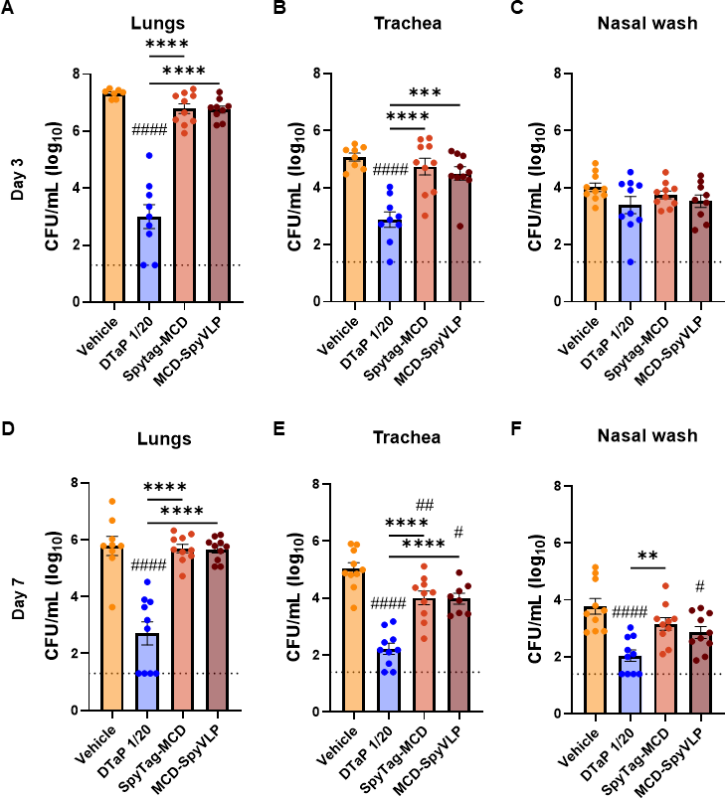
Vaccination with MCD-SpyVLP facilitates the control of bacterial burden in the airways of mice infected with *B. pertussis*. Bacterial burden in the lungs (**A and D**), trachea (**B and E**), and nasal wash (**C and F**) of mice immunized with SpyVLP-alone (vehicle), 1/20th human dose of DTaP, SpyTag-MCD, or MCD-SpyVLP was quantified at 3- (top) or 7-days (bottom) post-challenge with *B. pertussis* Tohama I. Horizontal dashed lines represent the lower limit of detection. *P*-values were calculated using ordinary one-way ANOVA with Tukey’s multiple comparisons test. *P < 0.05 and **P < 0.01. # represents comparisons to vehicle-immunized mice (*n* = 7–10 mice per group, bars represent mean ± SEM).

FHA was originally included in aP vaccines as it has been shown to contribute to vaccine-mediated protection against *B. pertussis* (44). *In vitro,* anti-FHA antibodies limit biofilm formation and have been shown to contribute to direct bacterial killing through agglutination (45). *In vivo* studies have also demonstrated that passive immunization with anti-FHA immune sera can limit infection in mice (29, 46). In addition, recombinantly produced regions of FHA that encompass the MCD are sufficient for protection against *B. pertussis* challenge in mice (28). To determine if SpyTag-MCD confers protection against infection with *B. pertussis,* mice were challenged intranasally with 2 × 10^6^ CFU of *B. pertussis* strain Tohama I 2 weeks after the boost. Bacterial burden was enumerated in the lungs, trachea, and nares of these mice post-euthanasia at days 3 and 7 following the challenge. Mice vaccinated with DTaP had a significantly lower bacterial burden in the lungs and trachea compared to mock-immunized animals 3 days after the challenge (Fig. 5A and B). No significant differences in bacterial burden in the nasal wash were detected in any groups by day 3 (Fig. 5C). At day 7 post-challenge, only DTaP-immunized mice had a reduction in bacterial burden in the lungs, trachea, and nasal wash (Fig. 5D). Interestingly, mice immunized with SpyTag-MCD and MCD-SpyVLP formulations had a lower bacterial burden in the trachea (Fig. 5E). In addition to lowering tracheal bacterial burden at day 7, MCD-SpyVLP provided enhanced protection in the nasal cavity compared to mock-immunized mice (Fig. 5F). These data suggest that immunization with MCD-SpyVLP confers protection against *B. pertussis* in mice, but that the protection it provides alone is insufficient to enable early clearance of the pathogen across the entire airway.

### Genetically inactive pertussis toxin-expressing *B. pertussis* infects mice with minimal neutrophilia and lung inflammation

**Fig 6:**
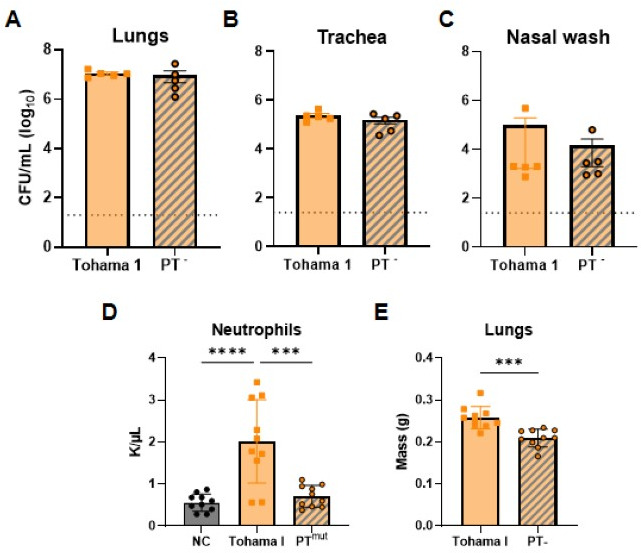
Genetically inactive pertussis toxin-expressing *B. pertussis* infects mice with minimal neutrophilia and lung inflammation. (**A**) Bacterial burden in the lungs (**A**), trachea (**B**), and nasal wash (**C**) 3 days post-challenge with *B. pertussis* Tohama I or Tohama I PT^mut^ (*n* = 10 mice per group, bars represent mean ± SEM). (**D**) Neutrophilia was quantified as neutrophils in the blood 3 days post-challenge in comparison to non-challenged (NC) mice. (**E**) Wet lung weights were measured 3 days after the challenge. *P*-values were determined using ordinary one-way ANOVA with Tukey’s multiple comparisons test. **P < 0.01 (bars represent mean ± SD).

Acellular pertussis vaccine formulations typically contain three to five antigens. Pertussis toxin is arguably the most important antigen in acellular formulations as it leads to the production of anti-pertussis toxin antibodies that neutralize toxin function and play a crucial role in protection against severe disease manifestation (47, 48). In the absence of neutralizing antibodies, PT can modulate and suppress host immune responses, leading to impaired pathogen clearance, reviewed in reference (49). We hypothesize that MCD-SpyVLP needs to be formulated alongside PT to generate the production of antibodies that both recognize the surface of the bacterium (anti-MCD antibodies) and neutralize PT (anti-PT antibodies) to provide complete protection. Our attempt to incorporate PT into our vaccine was hindered by two major factors. First, replicating the PT production, purification, and detoxification process within the laboratory setting presents substantial technological challenges, which were not feasible for this study. Second, stocks of genetically detoxified PT are no longer commercially available. To circumvent these technical issues, the efficacy of MCD-SpyVLP was tested in a model of *B. pertussis* challenge in which mice were challenged with a Tohama I derivative strain expressing genetically inactive PT (Tohama I PT^mut^) to mimic the effect of PT^mut^ neutralization by antibodies at the time of challenge. Challenge with Tohama I PT^mut^ was found to be similar to the challenge with the parental Tohama I strain, with similar levels of bacterial burden across the airway 3 days after the challenge (Fig. 6A through C). However, hallmark signs of PT activity, such as increased lung weight due to inflammation and neutrophilia, were significantly lower in mice challenged with Tohama I PT^mut^, consistent with the fact that this strain does not have active holotoxin due to the genetic inactivation (Fig. 6D and E).

### Vaccination with MCD-SpyVLP is protective against challenge with a strain expressing inactive pertussis toxin

**Fig 7:**
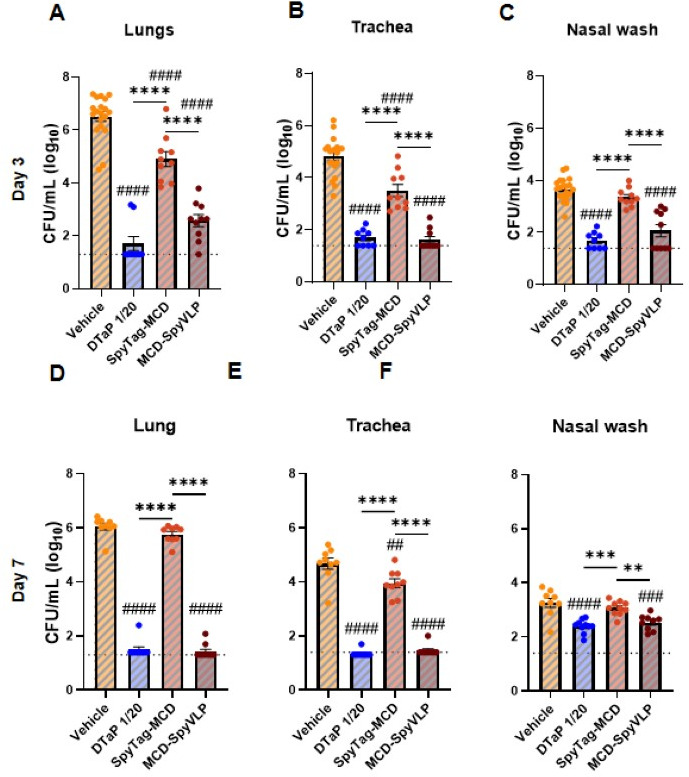
MCD-SpyVLP-mediated protection is enhanced without active pertussis toxin. Bacterial burden in the lungs (**A and D**), trachea (**B and E**), and nasal wash (**C and F**) of mice immunized with SpyVLP-alone (vehicle), 1/20th human doses of DTaP, SpyTag-MCD, or MCD-SpyVLP was quantified at 3- (top) or 7-days (bottom) post-challenge with *B. pertussis* Tohama I PT^−^. Horizontal dashed lines represent the lower limit of detection. *P*-values were calculated using ordinary one-way ANOVA with Tukey’s multiple comparisons test. **P < 0.01, ***P < 0.001, and ****P < 0.0001. # represents comparisons to vehicle-immunized mice (*n* = 9–10 mice per group, bars represent mean ± SEM).

To determine the protective efficacy of SpyTag-MCD in a context in which PT is inactive, a PT^mut^ strain of *B. pertussis* was used to challenge mice vaccinated with the schedule and formulations described above. Mice immunized with SpyTag-MCD had significantly reduced bacterial burden in the lungs and trachea compared to mock-immunized mice at both 3- and 7-days post-challenge. Expectedly, conjugation to SpyVLP significantly improved protection provided by MCD compared to mock immunization and SpyTag-MCD-vaccinated mice across the upper and the lower airways at both days 3 and 7 post-challenge (Fig. 7A through F). Remarkably, most MCD-SpyVLP vaccinated mice had undetectable CFUs in the lung and trachea by day 7, comparable to mice immunized with DTaP (Fig. 7D and E). These data suggest that in the absence of active PT, vaccination with MCD-SpyVLP provides significant protection in mice compared to SpyTag-MCD antigen alone, leading to near-complete clearance of the pathogen in the airways.

### Passive immunization with anti-MCD-SpyVLP immune sera provides protection against *B. pertussis* PT^mut^

**Fig 8:**
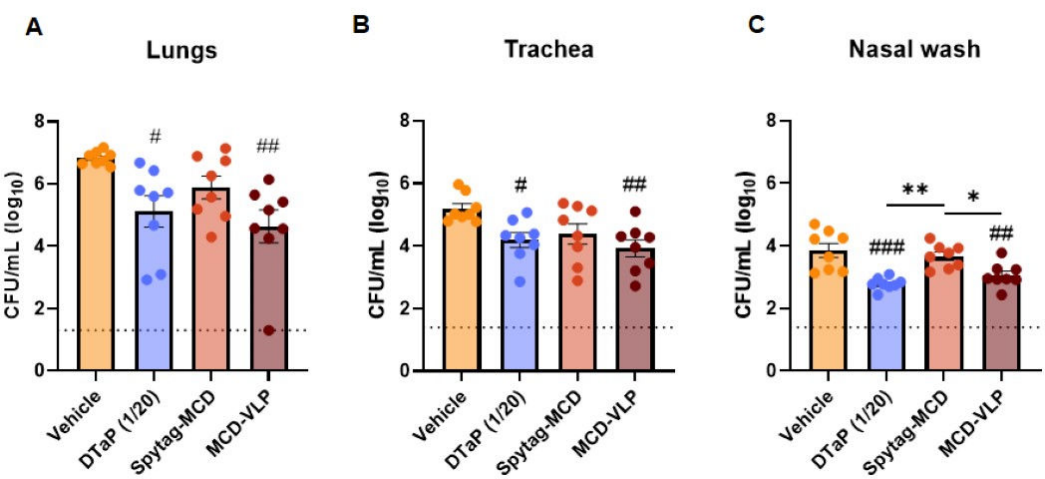
Passive immunization with anti-MCD-SpyVLP immune sera provides protection against *B. pertussis* PT^mut^ in the lungs. Bacterial burden in the lungs (**A**), trachea (**B**), and nasal wash (**C**) of mice passively immunized with SpyVLP-alone (vehicle), 1/20th DTaP Infanrix, SpyTag-MCD, or MCD-SpyVLP pooled sera was quantified at 3-days post-challenge with *B. pertussis* PT^mut^. *P*-values were calculated using ordinary one-way ANOVA with Tukey’s multiple comparisons test. *P < 0.05 and **P < 0.01. # represents comparisons to vehicle-immunized mice (*n* = 8 mice per group, bars represent mean ± SEM).

To determine if the protection provided by MCD-SpyVLP against the PT^mut^ strain is due to the presence of anti-*B*. *pertussis* antibodies in these mice, passive immunization experiments were conducted. Naive mice were intraperitoneally administered 250 µL of pooled sera extracted from SpyTag-MCD- or MCD-SpyVLP-immunized mice 1 week after boost. As controls, serum from mice immunized with SpyVLP (vehicle) or 1/20th a human dose DTaP Infanrix was used. Interestingly, mice passively immunized with DTaP or MCD-SpyVLP sera had decreased bacterial burden in the lungs, trachea, and nasal wash, whereas mice administered sera from Spytag-MCD-immunized mice did not (Fig. 8A through C). Overall, these data suggest that antibodies raised in response to MCD-SpyVLP immunization contribute to protection against the PT^mut^ strain across the airway, suggesting a functional role of anti-MCD antibodies in protection against *B. pertussis*.

## DISCUSSION

To combat the persisting public health challenge that *B. pertussis* poses in many countries, it is increasingly important to develop pertussis vaccines that are highly immunogenic and target protective regions within pertussis antigens. Additionally, it is crucial to also make these vaccines as cost-effective as possible. Despite its inclusion in all multi-subunit aPs, FHA’s role in eliciting protective immunity remains speculative, despite *in vitro* and preclinical evidence demonstrating that anti-FHA antibodies can limit bacterial growth and provide protection against *B. pertussis* infection (45, 46). In this study, the protective efficacy of a truncated FHA molecule consisting of amino acids 1,871–2,362 of mature FhaB (termed the MCD) was evaluated as a vaccine antigen against *B pertussis*. This region plays a critical role in the export of FHA onto the bacterial surface, is involved in bacterial biofilm formation, and may contribute to FHA-mediated protection given its immunodominance and availability to immune cells as it is distal to the bacterial surface (22, 25, 50). The MCD of FHA is also highly soluble and can be produced efficiently in *E. coli*, which could facilitate production and reduce the cost of producing an FHA-based antigen for inclusion in aP vaccines.

Here, it was demonstrated that the same mass per dosage of MCD when conjugated to SpyCatcher003-mi3 VLPs leads to an overall enhanced response, generating levels of anti-*B*. *pertussis* antibodies that are comparable to DTaP, which includes the same mass of FHA and additional pertussis antigens. This enhanced immunogenicity is likely due, in part, to the greater size of the VLP particles and the repetitive nature of antigen valency when presented on VLPs, which allows for stronger and earlier B cell stimulation. In addition, by using the SpyTag-SpyCatcher platform, MCD is covalently bound to the VLP, which we hypothesize should allow for SpyTag-MCD-specific B cells to be stimulated by T cells, which recognize either MCD or the VLP itself (51). Unfortunately, direct comparison between FHA and MCD is challenging due to the presence of high levels of endotoxin in FHA, which in some cases can exceed 40,000 EU/mL and acts as a confounding factor when comparing antigen immunogenicity and efficacy (52). Studies determining whether vaccination with MCD-SpyVLP is superior or equivalent to vaccination with FHA would need to be performed with endotoxin-free FHA, which is currently unavailable and technically challenging to produce.

In this study, SpyTag-MCD and MCD-SpyVLP provided significant, yet insufficient protection against *B. pertussis* in the presence of PT. This is consistent with previous studies in which despite MCD immunogenicity, truncated FHA molecules have consistently been shown to only provide moderate control of bacterial burden in the upper or lower airway following challenge with *B. pertussis* (28, 29, 46). Similar results have been seen despite often using additional boosting events, antigen doses as high as 15 µg/dose, or lower challenge numbers of *B. pertussis* for the challenge (28, 29, 46).

While FHA is a primary component of most acellular pertussis vaccines, all aP vaccine formulations contain PT, which is both essential and sufficient to provide protection against *B. pertussis* (53, 54). PT is an AB_5_ multi-subunit protein toxin and is one of the most important pertussis virulence factors that has a breadth of immunosuppressive effects within the respiratory tract and throughout the body. Namely, by inhibiting G-protein signaling, PT reduces immune cell chemotaxis, impairs phagocytosis, and dysregulates cytokine production among other detrimental effects, all of which are critical for mounting an effective immune response (49). By utilizing the *B. pertussis* Tohama I PT^mut^ strain in this study, the objective was to understand how anti-MCD responses are impacted by PT toxicity and how MCD-SpyVLP functions in the context of a vaccine that effectively inhibits PT. Here, both MCD formulations were demonstrated to provide enhanced protection against Tohama I PT^mut^ early after the challenge compared to mock-immunized animals, whereas MCD-SpyVLP outperformed SpyTag-MCD across the entire airway. Interestingly, by day 7, most mice immunized with MCD-SpyVLP had undetectable CFUs in the lungs as well as trachea, whereas mice immunized with SpyTag-MCD no longer had lower bacterial burden than the mock-immunized group, suggesting that SpyTag-MCD was not sufficient to maintain bacterial clearance in these organs. Additionally, only MCD-SpyVLP provided reduced bacterial burden in the nares at day 3 or 7. Overall, these data suggest that PT must be inhibited to observe an effective anti-MCD immune response and that linkage of MCD to VLPs is necessary to maintain bacterial clearance later during infection.

While it was demonstrated that MCD can be a protective antigen against *B. pertussis,* the specific role that anti-MCD antibodies play in protection following challenge with the PT^mut^ strain was also evaluated. Here, it was demonstrated that passive immunization with immune sera pooled from MCD-SpyVLP-immunized mice can limit infection in the lungs of mice, whereas serum from SpyTag-MCD-immunized animals cannot. This suggests that the quantity of antibodies present may be a key factor in limiting infection. Interestingly, meanwhile, DTaP-immunized mice maintain similar levels of anti-FHA and MCD antibodies, mice given sera from these animals did not have similar protection in the lungs compared to MCD-SpyVLP. A potential reason for this is the loss of protective FHA epitopes that may occur through formaldehyde treatment during vaccine manufacturing, as has been repeatedly demonstrated with chemically denatured pertussis toxin (13 – 15). Indeed, the peptide epitope mapping analysis supports this hypothesis as antibodies in pooled sera from DTaP-immunized mice bind fewer epitopes within the MCD region of FHA. It is important to note, however, that this analysis was limited to *in silico*-predicted linear epitopes and does not account for conformational epitopes. Likewise, MCD-SpyVLP may provide enhanced protection through the increased breadth of MCD epitope recognition. While quantifying and determining the effect of antibodies was a key focus of this work, FHA is a dominant target of anti-*B*. *pertussis* T cell responses following infection as well as vaccination, and it is possible that T cells elicited against MCD also contribute to the protection (55 – 57). Additionally, the peptide epitope mapping is not without caveats, such as differences in peptide binding depending on the ELISA and the use of only linear B cell epitopes. To determine more precise regions within MCD that are being bound by antibodies, further studies using cryo-electron microscopy would be necessary.

The improved efficacy of MCD through conjugation to VLPs is crucial as it has the potential to lead to the development of protective vaccines that can be more easily produced in *E. coli*. While MCD was used as a proof-of-concept antigen in this study, this same approach could be taken for other pertussis antigens as it has been done for other pathogens (30, 34, 40). Future studies evaluating the protective efficacy of MCD-SpyVLP formulated either alone or with different adjuvants could contribute to further increasing the number of antigen-specific ASC and the memory response to aP vaccines.

It is important to acknowledge that the use of a pertussis toxin mutant strain in our studies may not fully replicate the conditions of vaccination with PT-containing vaccines or natural infection with PT-expressing *B. pertussis* strains. While the PT mutant strain allowed an understanding of the immune responses to the MCD without the confounding effects of PT’s immunosuppressive properties, it does not entirely capture the complex interactions that occur in the presence of PT. Additionally, the efficacy of MCD-SpyVLP against currently circulating clinical isolates remains undetermined; however, our data indicate that antibodies generated in these mice do bind to various isolates, suggesting potential cross-protection (Fig. S1). Future studies will aim to evaluate the efficacy of MCD-SpyVLP in conjunction with PT against currently circulating clinical isolates and evaluate sex as a biological variable in the response to MCD-SpyVLP vaccination.

In conclusion, this study introduces a novel approach to pertussis vaccination that utilizes virus-like particles to enhance the immunogenicity of already established pertussis antigens while targeting critical domains within the antigens themselves. This strategy not only enhanced the immunogenicity of MCD as an antigen but could also increase the longevity of anti-*B*. *pertussis* immune responses while using a highly scalable and cost-effective antigen production method. Additionally, this study provided a proof-of-principle that anti-MCD antibodies alone can contribute to protection against *B. pertussis* and that anti-MCD immunity is only effective in the absence of active PT, which should be taken into consideration when studying other novel pertussis antigens for inclusion in vaccines, as many single antigen vaccines against *B. pertussis* are ineffective without the inclusion of PT. This lays the groundwork for future studies to explore the full potential of the SpyVLP platform for use in the development of pertussis vaccines.

## SUPPLEMENTAL MATERIAL

Supplemental material iai.00270-24-s0001.docx Figure S1 and supplemental methods.

ASM does not own the copyrights to Supplemental Material that may be linked to, or accessed through, an article. The authors have granted ASM a non-exclusive, world-wide license to publish the Supplemental Material files. Please contact the corresponding author directly for reuse.
