# Supplementary material for: Virus-like particles displaying the mature C-terminal domain of filamentous hemagglutinin are immunogenic and protective against Bordetella pertussis respiratory infection in mice
Source: Infect Immun. 2024 Jul 18;92(8):e00270-24. doi: 10.1128/iai.00270-24 (PMC11320929; doi:10.1128/iai.00270-24)
Supplement: Supplemental material — Figure S1 and supplemental methods. [file iai.00270-24-s0001.docx]

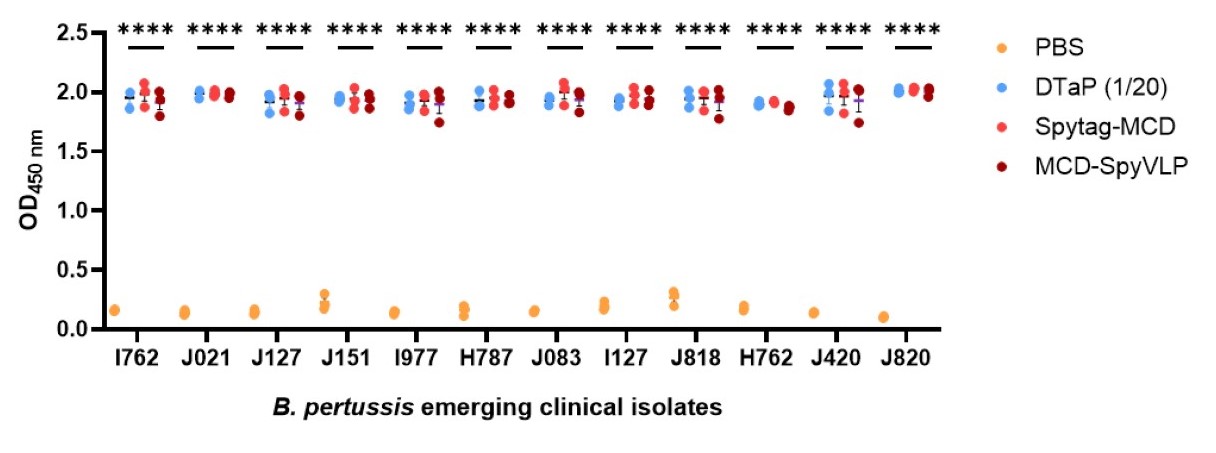


**Supplementary Figure 1: SpyTag-MCD and MCD-SpyVLP generate serum IgG that broadly recognize emerging clinical isolates of *B. pertussis*.** ELISA was used to detect binding of sera pooled from mice (n=5) immunized with 1/20th human dose of DTaP, FHA, SpyTag-MCD, or MCD-SpyVLP to 12 emerging clinical isolates of *B. pertussis*. *p*-values were calculated using ordinary one-way ANOVA with Tukey’s multiple comparisons test for each isolate. *p*-values represent significant differences compared to vehicle-immunized mice (PBS). *****p* < 0.0001

**Supplemental Methods**

***Quantification of antibody binding to B. pertussis emerging clinical isolates:***

*B. pertussis* emerging clinical isolates were provided by the Centers for Disease Control and Prevention. All *B. pertussis* strains were cultured on Bordet-Gengou agar (VWR™, Cat. #90003–41) supplemented with 15% defibrinated sheep’s blood (Hemostat Laboratories, Cat. #DSB500) and 40 µg/mL cephalexin (Sigma-Aldrich, Cat. #C4895) for 48 h at 36^°^C. Strains were then collected using sterile polyester swabs (Puritan, Cat. #22-029-574) and transferred into 20 mL of sterile Stainer-Scholte liquid media in 125 mL flasks at 36^°^C with constant agitation at 180 rpm until reaching an OD_600nm_ with 1 cm path width of 0.4-0.5 (10). Liquid cultures were diluted to an OD_600nm_ of 0.245 corresponding to 10^9^ CFU/mL and used to coat high-binding ELISA plates (ThermoFisher, Cat. #15041) (50 µL/well) overnight at 4ºC. After coating, plates were blocked overnight using 200 µL/well of 5% nonfat dry milk (Nestle Carnation, Cat. #00500002292840) in PBS-tween 20 (PBS-T). Plates were then washed with PBS-T, and pooled sera (n=5 per group) were prepared at a dilution of 1:1,000 in 5% nonfat dry milk in PBS-T and added to wells containing individual isolates (n=3 per isolate) and incubated for 2 h at 37^°^C. Plates were then washed and incubated with 100 µL/well goat anti-mouse IgG horse-radish peroxidase-conjugated (HRP) antibodies (Novus Biologicals, Cat. #NBP1-75130) diluted 1:2,000 for 1 h at 37^°^C. Plates were then washed and incubated with 100 µL tetramethylbenzidine (TMB) substrate (BioLegend, Cat. #421101) for 30 min at room-temperature covered from light. After 30 min, 50 µL of 2 M sulfuric acid (Fisher Scientific, Cat. #SA818500) was added to stop the reaction. Absorbance at OD_450_ _nm_ was then read using a SpectraMax i3 plate reader (Molecular Devices).
